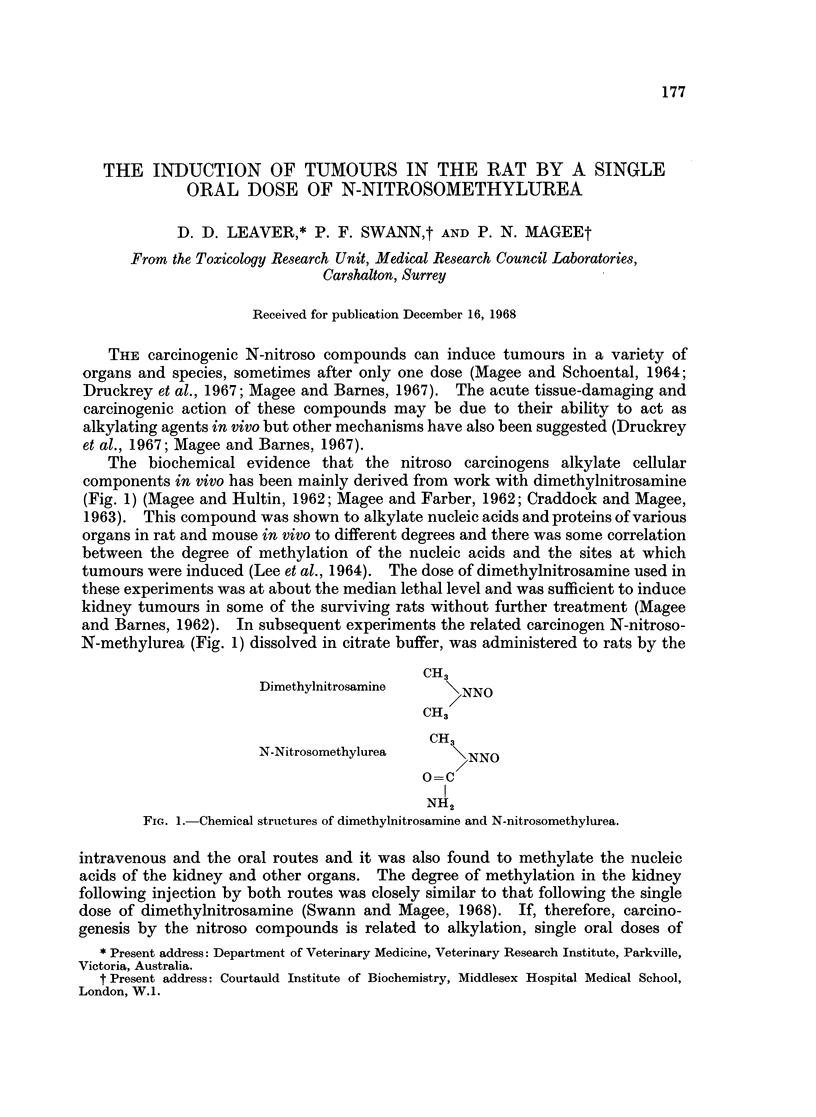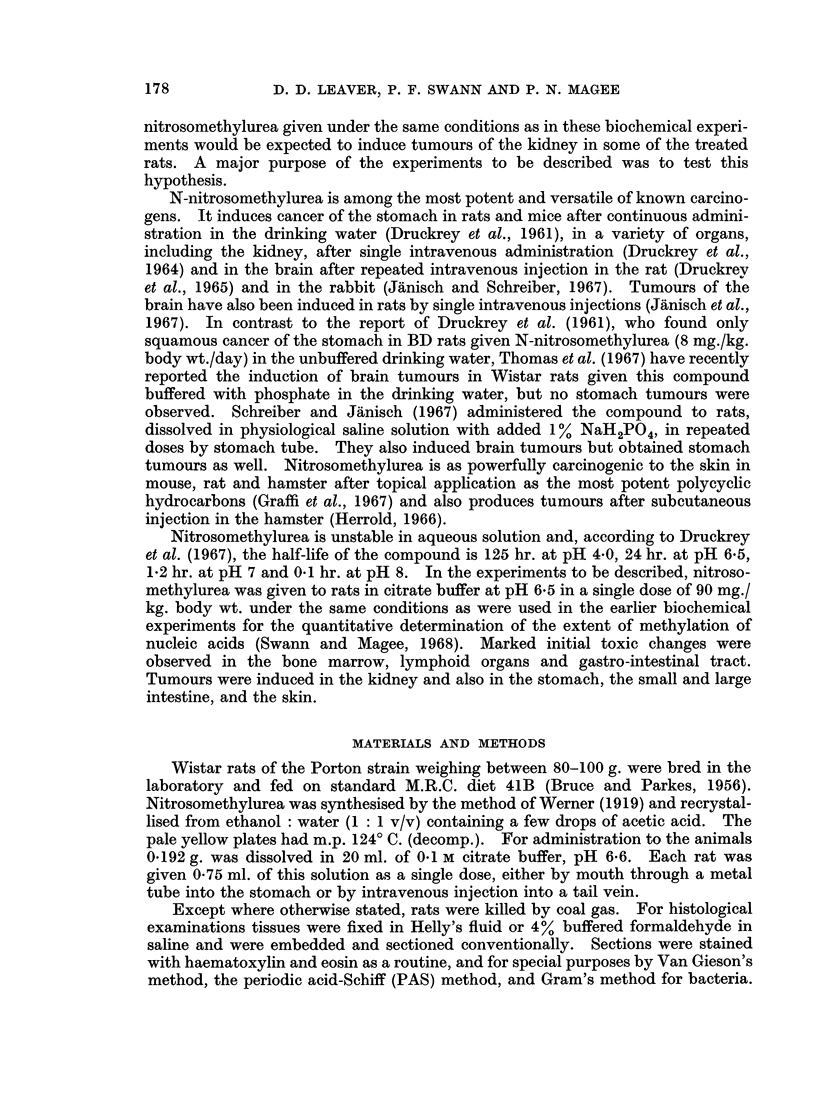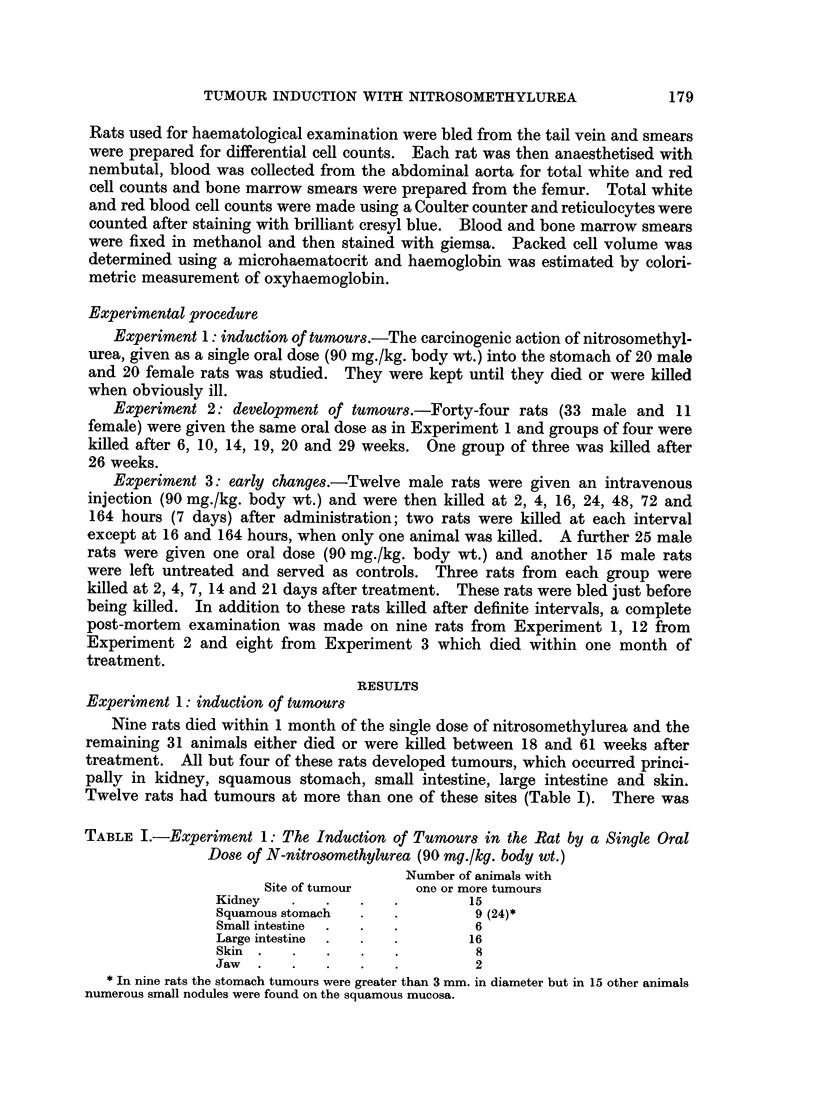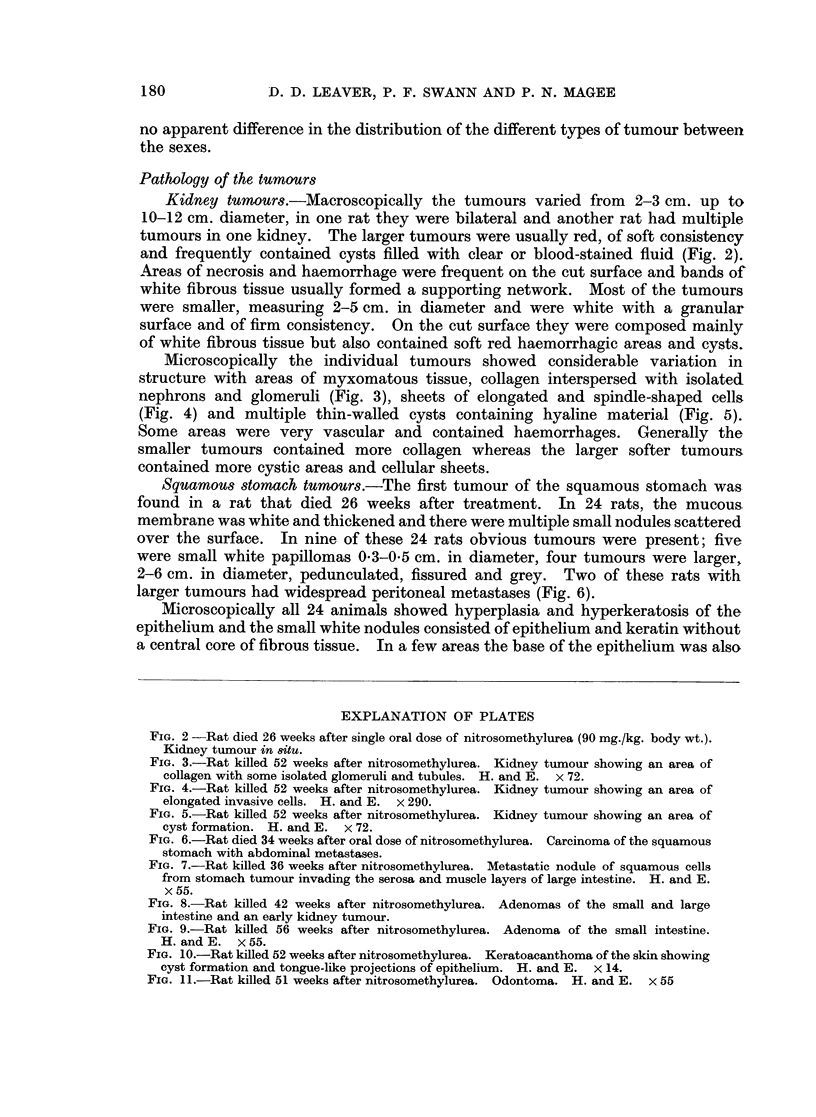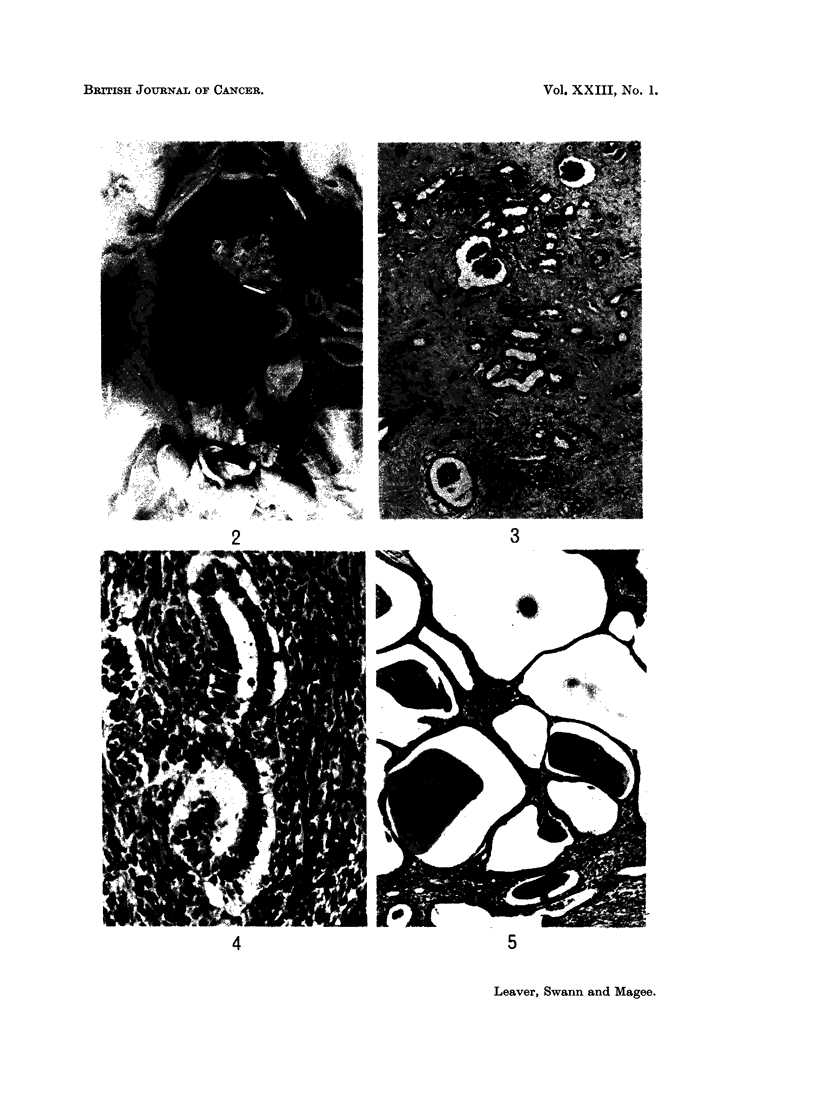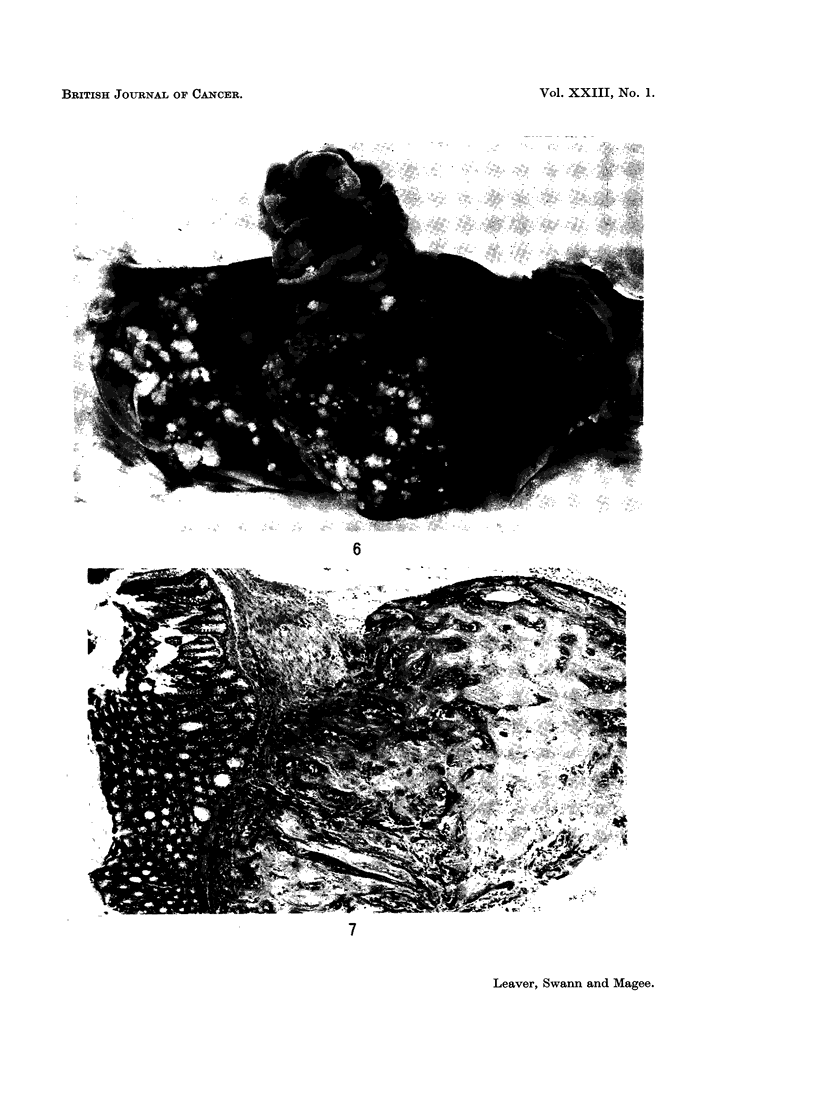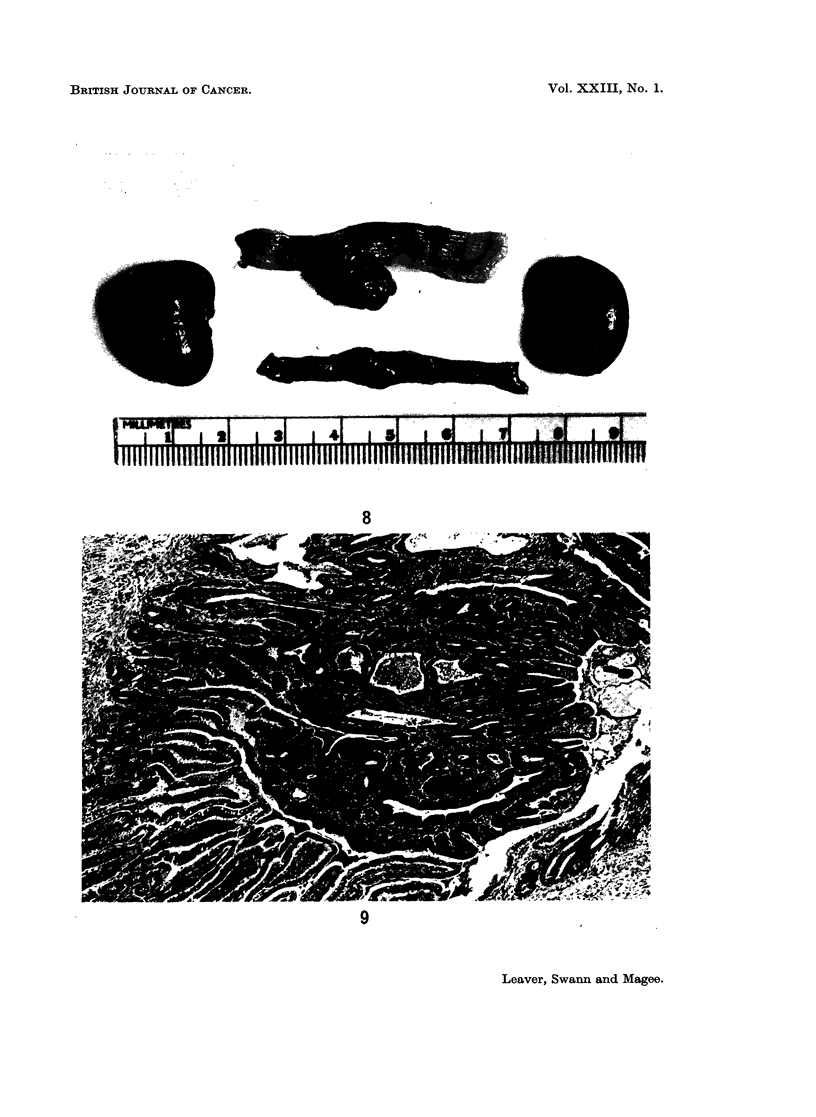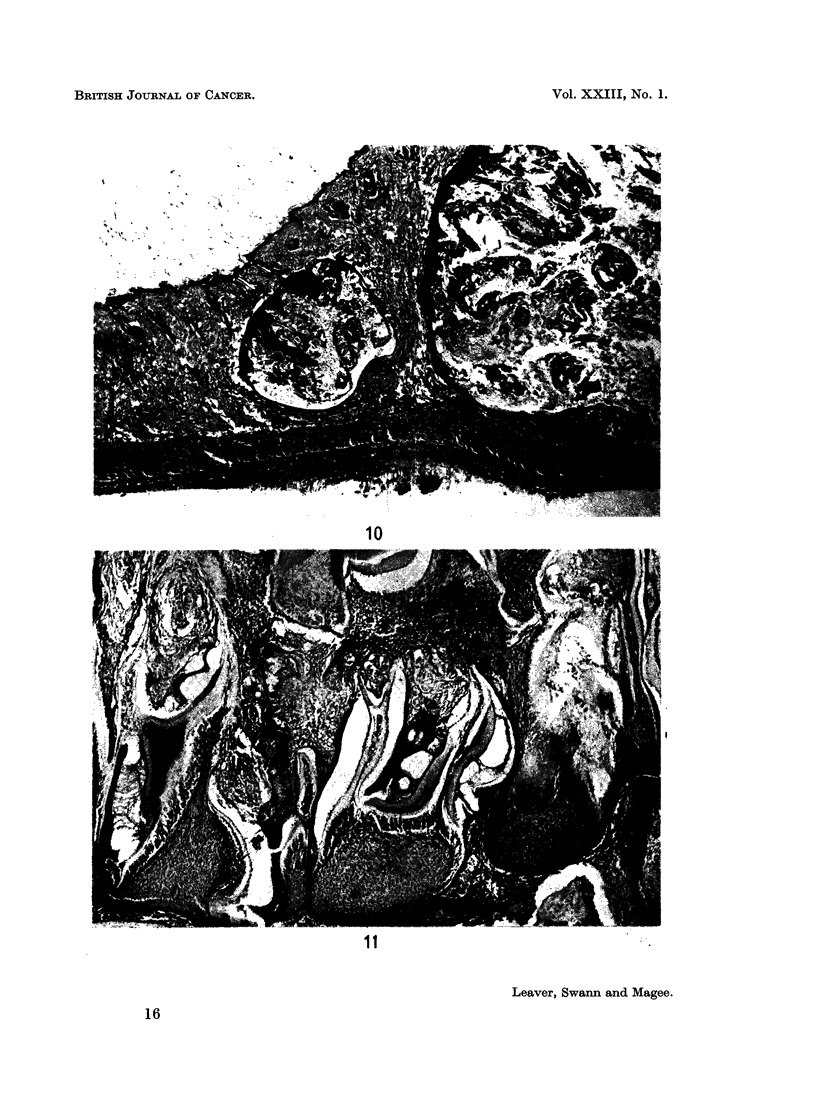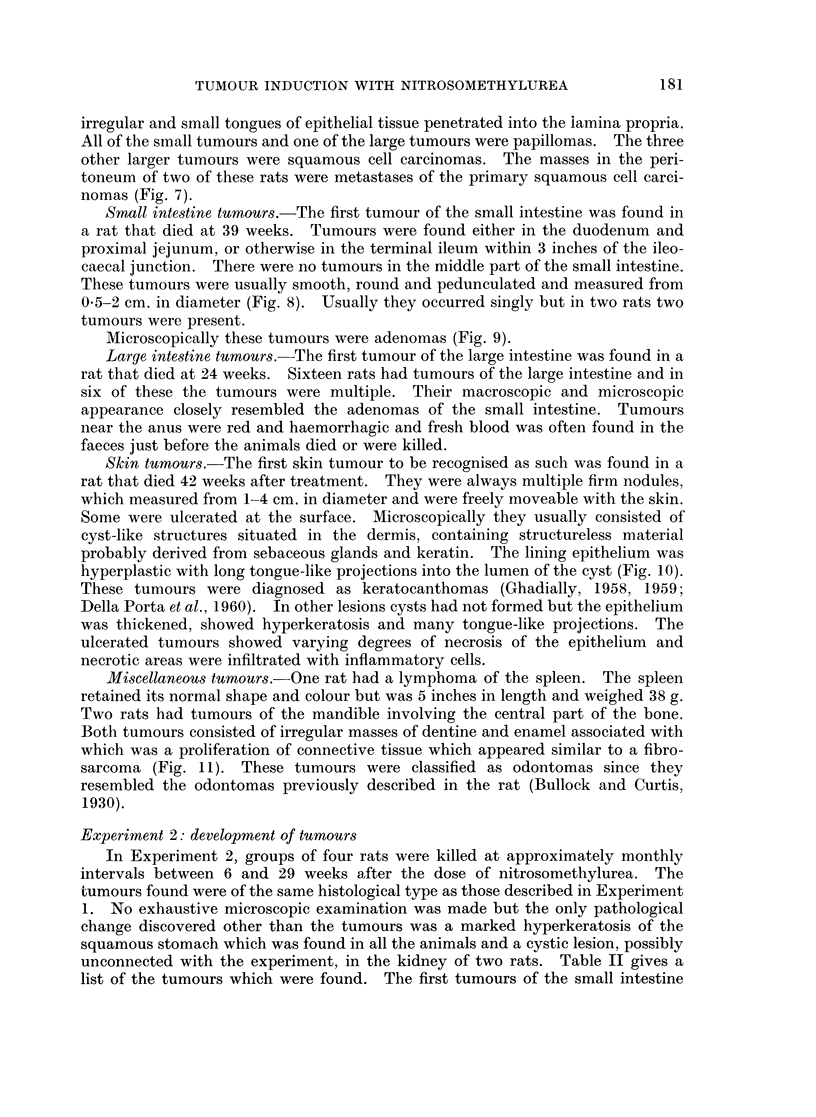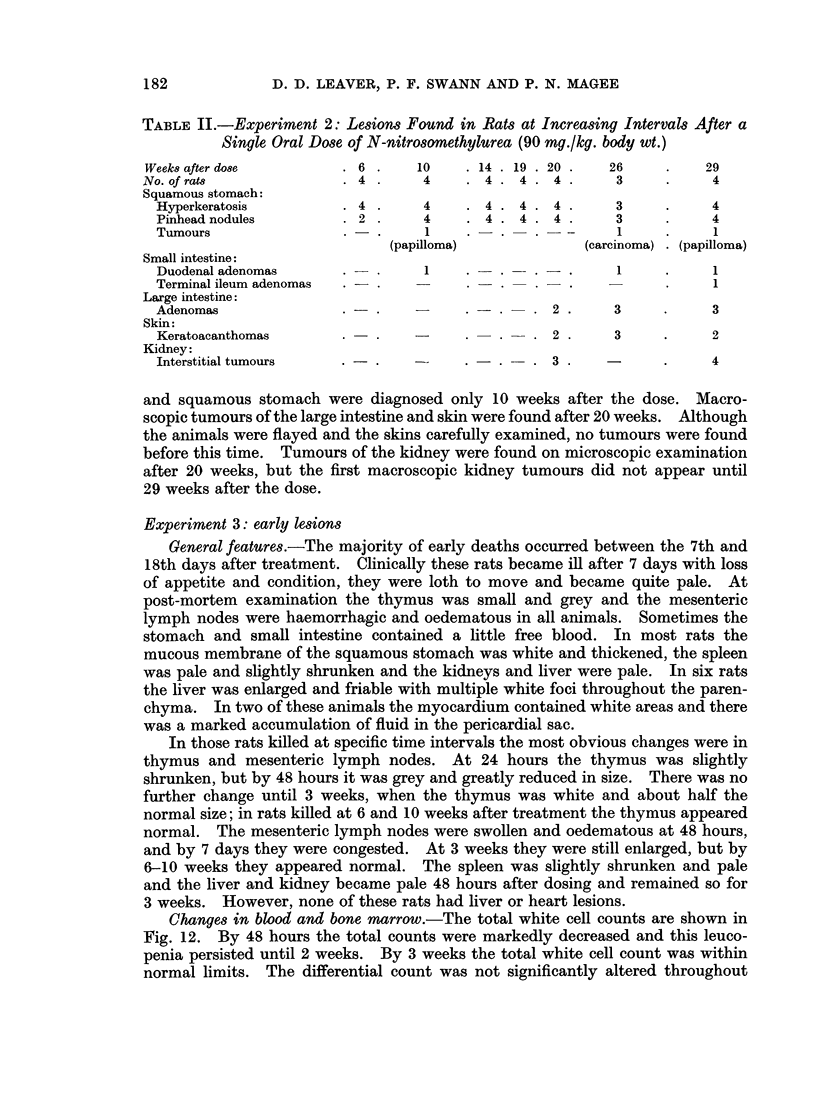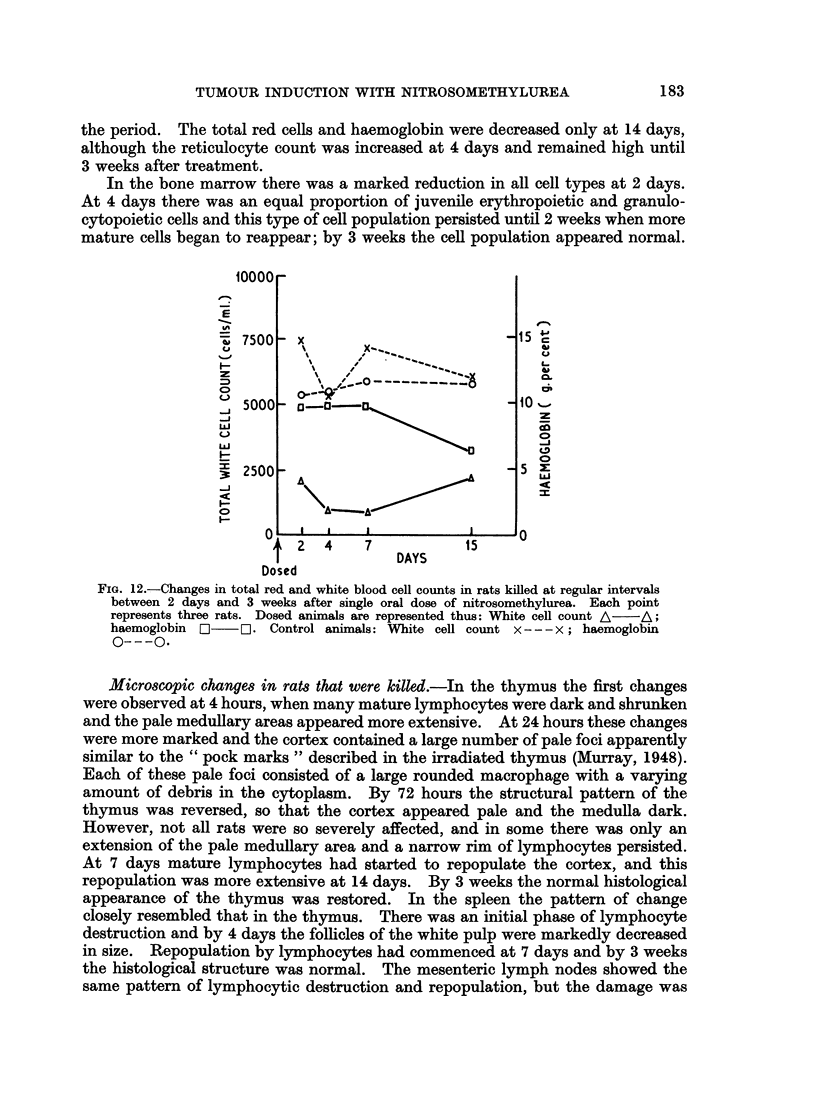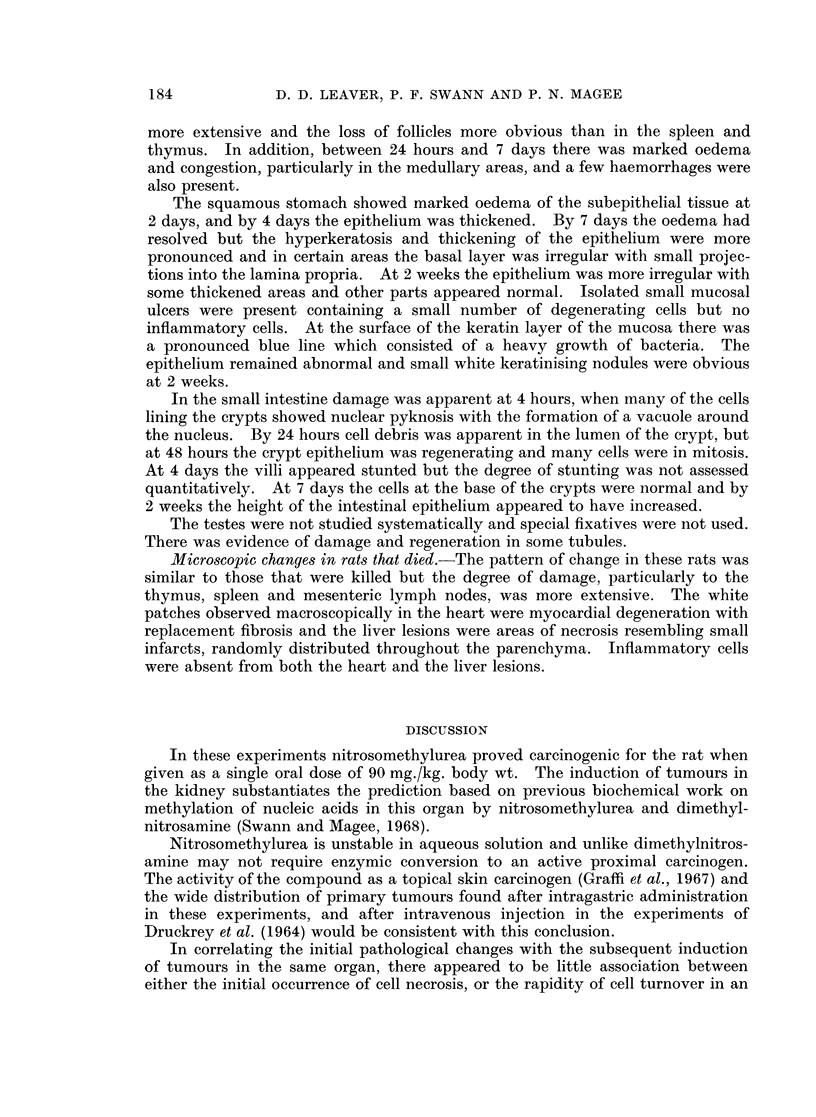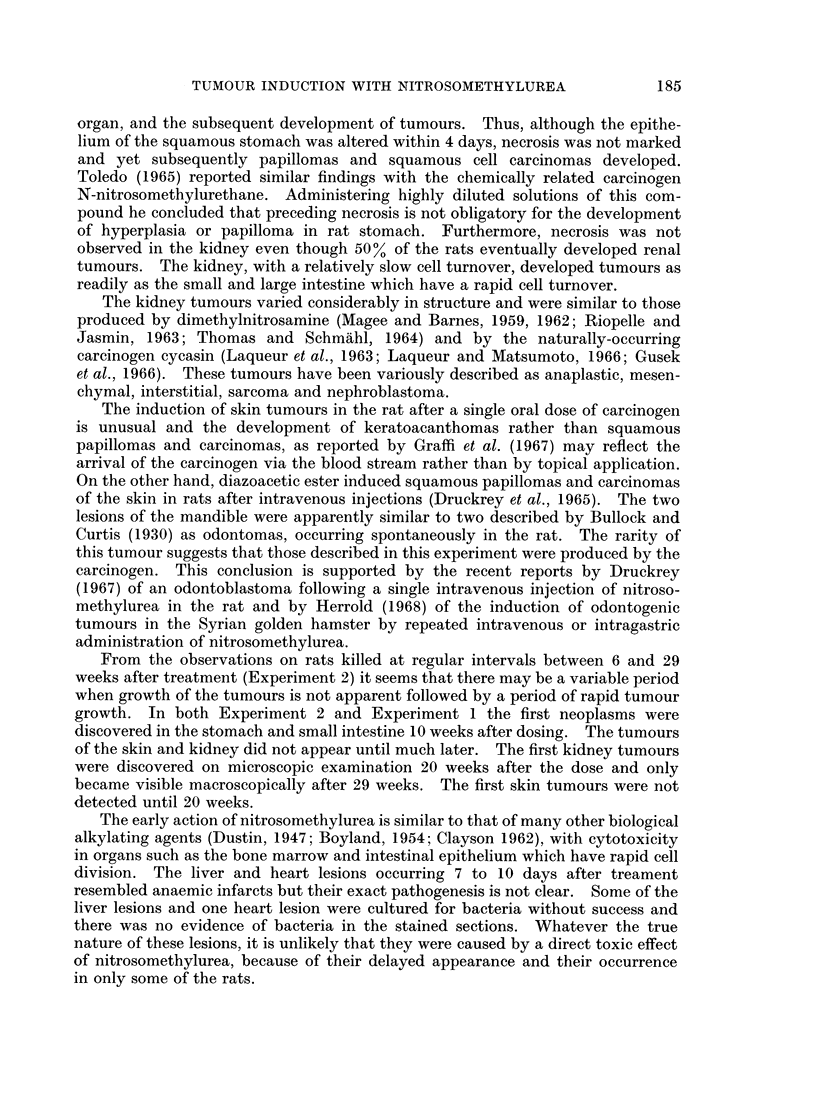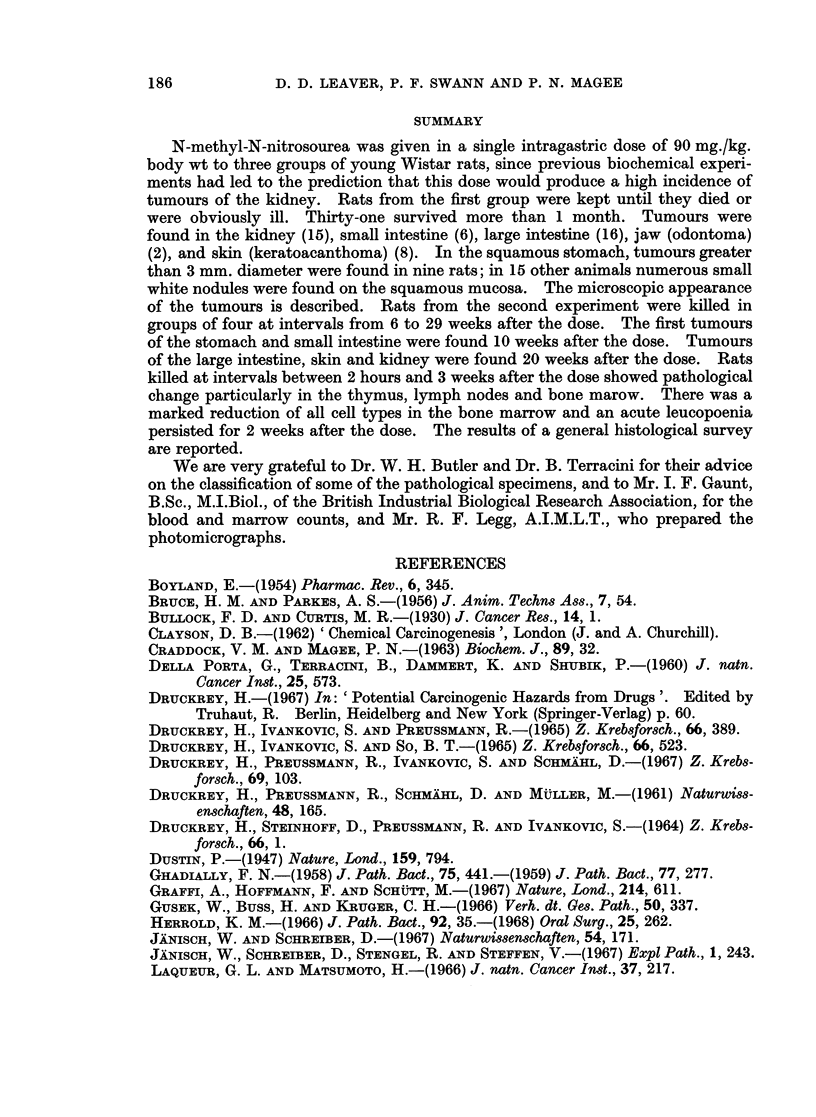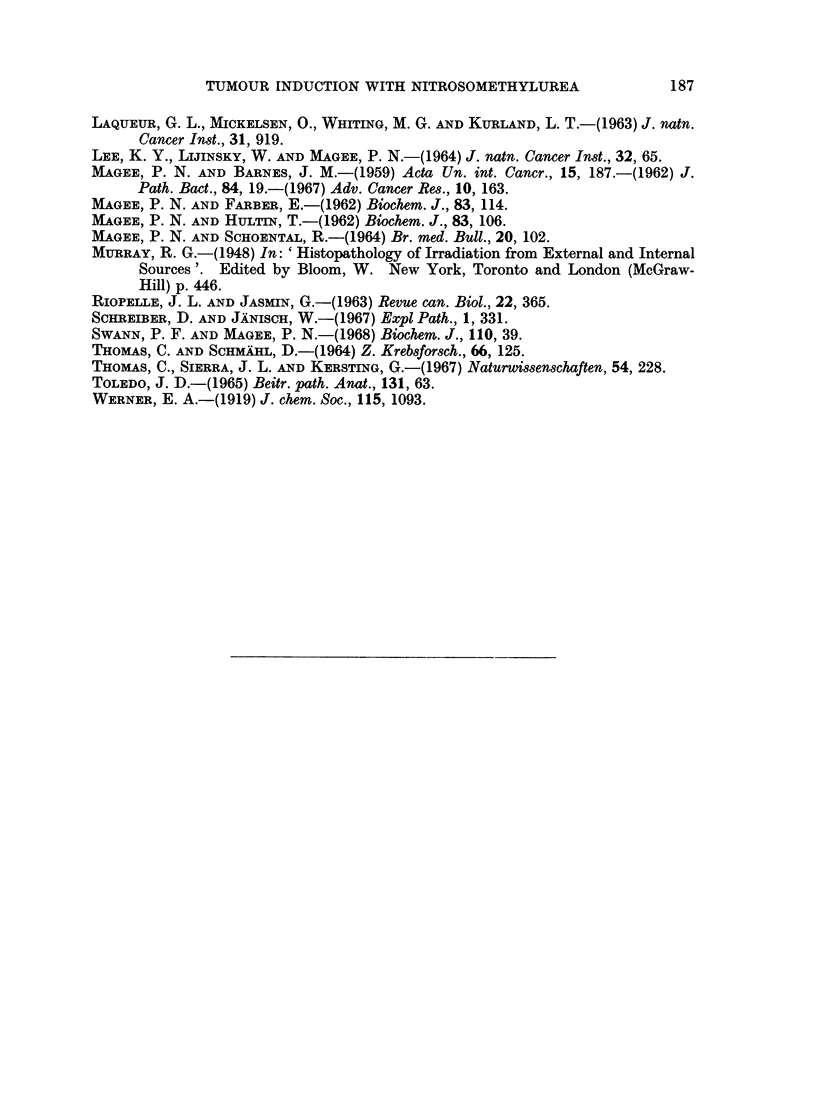# The induction of tumours in the rat by a single oral dose of N-nitrosomethylurea.

**DOI:** 10.1038/bjc.1969.26

**Published:** 1969-03

**Authors:** D. D. Leaver, P. F. Swann, P. N. Magee

## Abstract

**Images:**


### THE INDUCTION OF TUMOURS IN THE RAT BY A SINGLE ORAL DOSE OF N-NITROSOMETHYLUREA

D. D. LEAVER,* P. F. SWANN,† AND P. N. MAGEE†

*From the Toxicology Research Unit, Medical Research Council Laboratories, Carshalton, Surrey*

Received for publication December 16, 1968

THE carcinogenic N-nitroso compounds can induce tumours in a variety of organs and species, sometimes after only one dose (Magee and Schoental, 1964; Druckrey *et al.*, 1967; Magee and Barnes, 1967). The acute tissue-damaging and carcinogenic action of these compounds may be due to their ability to act as alkylating agents *in vivo* but other mechanisms have also been suggested (Druckrey *et al.*, 1967; Magee and Barnes, 1967).

The biochemical evidence that the nitroso carcinogens alkylate cellular components *in vivo* has been mainly derived from work with dimethylnitrosamine (Fig. 1) (Magee and Hultin, 1962; Magee and Farber, 1962; Craddock and Magee, 1963). This compound was shown to alkylate nucleic acids and proteins of various organs in rat and mouse *in vivo* to different degrees and there was some correlation between the degree of methylation of the nucleic acids and the sites at which tumours were induced (Lee *et al.*, 1964). The dose of dimethylnitrosamine used in these experiments was at about the median lethal level and was sufficient to induce kidney tumours in some of the surviving rats without further treatment (Magee and Barnes, 1962). In subsequent experiments the related carcinogen N-nitroso-N-methylurea (Fig. 1) dissolved in citrate buffer, was administered to rats by the

Dimethylnitrosamine

N-Nitrosomethylurea

FIG. 1.—Chemical structures of dimethylnitrosamine and N-nitrosomethylurea.

intravenous and the oral routes and it was also found to methylate the nucleic acids of the kidney and other organs. The degree of methylation in the kidney following injection by both routes was closely similar to that following the single dose of dimethylnitrosamine (Swann and Magee, 1968). If, therefore, carcinogenesis by the nitroso compounds is related to alkylation, single oral doses of

* Present address: Department of Veterinary Medicine, Veterinary Research Institute, Parkville, Victoria, Australia.

† Present address: Courtauld Institute of Biochemistry, Middlesex Hospital Medical School, London, W.1.

nitrosomethylurea given under the same conditions as in these biochemical experiments would be expected to induce tumours of the kidney in some of the treated rats. A major purpose of the experiments to be described was to test this hypothesis.

N-nitrosomethylurea is among the most potent and versatile of known carcinogens. It induces cancer of the stomach in rats and mice after continuous administration in the drinking water (Druckrey *et al.*, 1961), in a variety of organs, including the kidney, after single intravenous administration (Druckrey *et al.*, 1964) and in the brain after repeated intravenous injection in the rat (Druckrey *et al.*, 1965) and in the rabbit (Jänisch and Schreiber, 1967). Tumours of the brain have also been induced in rats by single intravenous injections (Jänisch *et al.*, 1967). In contrast to the report of Druckrey *et al.* (1961), who found only squamous cancer of the stomach in BD rats given N-nitrosomethylurea (8 mg./kg. body wt./day) in the unbuffered drinking water, Thomas *et al.* (1967) have recently reported the induction of brain tumours in Wistar rats given this compound buffered with phosphate in the drinking water, but no stomach tumours were observed. Schreiber and Jänisch (1967) administered the compound to rats, dissolved in physiological saline solution with added 1% $NaH_2PO_4$, in repeated doses by stomach tube. They also induced brain tumours but obtained stomach tumours as well. Nitrosomethylurea is as powerfully carcinogenic to the skin in mouse, rat and hamster after topical application as the most potent polycyclic hydrocarbons (Graffi *et al.*, 1967) and also produces tumours after subcutaneous injection in the hamster (Herrold, 1966).

Nitrosomethylurea is unstable in aqueous solution and, according to Druckrey *et al.* (1967), the half-life of the compound is 125 hr. at pH 4·0, 24 hr. at pH 6·5, 1·2 hr. at pH 7 and 0·1 hr. at pH 8. In the experiments to be described, nitrosomethylurea was given to rats in citrate buffer at pH 6·5 in a single dose of 90 mg./kg. body wt. under the same conditions as were used in the earlier biochemical experiments for the quantitative determination of the extent of methylation of nucleic acids (Swann and Magee, 1968). Marked initial toxic changes were observed in the bone marrow, lymphoid organs and gastro-intestinal tract. Tumours were induced in the kidney and also in the stomach, the small and large intestine, and the skin.

### MATERIALS AND METHODS

Wistar rats of the Porton strain weighing between 80–100 g. were bred in the laboratory and fed on standard M.R.C. diet 41B (Bruce and Parkes, 1956). Nitrosomethylurea was synthesised by the method of Werner (1919) and recrystallised from ethanol : water (1 : 1 v/v) containing a few drops of acetic acid. The pale yellow plates had m.p. 124° C. (decomp.). For administration to the animals 0·192 g. was dissolved in 20 ml. of 0·1 M citrate buffer, pH 6·6. Each rat was given 0·75 ml. of this solution as a single dose, either by mouth through a metal tube into the stomach or by intravenous injection into a tail vein.

Except where otherwise stated, rats were killed by coal gas. For histological examinations tissues were fixed in Helly's fluid or 4% buffered formaldehyde in saline and were embedded and sectioned conventionally. Sections were stained with haematoxylin and eosin as a routine, and for special purposes by Van Gieson's method, the periodic acid-Schiff (PAS) method, and Gram's method for bacteria.

Rats used for haematological examination were bled from the tail vein and smears were prepared for differential cell counts. Each rat was then anaesthetised with nembutal, blood was collected from the abdominal aorta for total white and red cell counts and bone marrow smears were prepared from the femur. Total white and red blood cell counts were made using a Coulter counter and reticulocytes were counted after staining with brilliant cresyl blue. Blood and bone marrow smears were fixed in methanol and then stained with giemsa. Packed cell volume was determined using a microhaematocrit and haemoglobin was estimated by colorimetric measurement of oxyhaemoglobin.

*Experimental procedure*

*Experiment* 1: *induction of tumours.*—The carcinogenic action of nitrosomethylurea, given as a single oral dose (90 mg./kg. body wt.) into the stomach of 20 male and 20 female rats was studied. They were kept until they died or were killed when obviously ill.

*Experiment* 2: *development of tumours.*—Forty-four rats (33 male and 11 female) were given the same oral dose as in Experiment 1 and groups of four were killed after 6, 10, 14, 19, 20 and 29 weeks. One group of three was killed after 26 weeks.

*Experiment* 3: *early changes.*—Twelve male rats were given an intravenous injection (90 mg./kg. body wt.) and were then killed at 2, 4, 16, 24, 48, 72 and 164 hours (7 days) after administration; two rats were killed at each interval except at 16 and 164 hours, when only one animal was killed. A further 25 male rats were given one oral dose (90 mg./kg. body wt.) and another 15 male rats were left untreated and served as controls. Three rats from each group were killed at 2, 4, 7, 14 and 21 days after treatment. These rats were bled just before being killed. In addition to these rats killed after definite intervals, a complete post-mortem examination was made on nine rats from Experiment 1, 12 from Experiment 2 and eight from Experiment 3 which died within one month of treatment.

### RESULTS

*Experiment* 1: *induction of tumours*

Nine rats died within 1 month of the single dose of nitrosomethylurea and the remaining 31 animals either died or were killed between 18 and 61 weeks after treatment. All but four of these rats developed tumours, which occurred principally in kidney, squamous stomach, small intestine, large intestine and skin. Twelve rats had tumours at more than one of these sites (Table I). There was

TABLE I.—*Experiment* 1: *The Induction of Tumours in the Rat by a Single Oral Dose of N-nitrosomethylurea* (90 *mg./kg. body wt.*)

| Site of tumour | Number of animals with one or more tumours |
|---|---|
| Kidney . . . . | 15 |
| Squamous stomach . . | 9 (24)* |
| Small intestine . . . | 6 |
| Large intestine . . . | 16 |
| Skin . . . . . . | 8 |
| Jaw . . . . . . | 2 |

* In nine rats the stomach tumours were greater than 3 mm. in diameter but in 15 other animals numerous small nodules were found on the squamous mucosa.

no apparent difference in the distribution of the different types of tumour between the sexes.

*Pathology of the tumours*

*Kidney tumours.*—Macroscopically the tumours varied from 2–3 cm. up to 10–12 cm. diameter, in one rat they were bilateral and another rat had multiple tumours in one kidney. The larger tumours were usually red, of soft consistency and frequently contained cysts filled with clear or blood-stained fluid (Fig. 2). Areas of necrosis and haemorrhage were frequent on the cut surface and bands of white fibrous tissue usually formed a supporting network. Most of the tumours were smaller, measuring 2–5 cm. in diameter and were white with a granular surface and of firm consistency. On the cut surface they were composed mainly of white fibrous tissue but also contained soft red haemorrhagic areas and cysts.

Microscopically the individual tumours showed considerable variation in structure with areas of myxomatous tissue, collagen interspersed with isolated nephrons and glomeruli (Fig. 3), sheets of elongated and spindle-shaped cells (Fig. 4) and multiple thin-walled cysts containing hyaline material (Fig. 5). Some areas were very vascular and contained haemorrhages. Generally the smaller tumours contained more collagen whereas the larger softer tumours contained more cystic areas and cellular sheets.

*Squamous stomach tumours.*—The first tumour of the squamous stomach was found in a rat that died 26 weeks after treatment. In 24 rats, the mucous membrane was white and thickened and there were multiple small nodules scattered over the surface. In nine of these 24 rats obvious tumours were present; five were small white papillomas 0·3–0·5 cm. in diameter, four tumours were larger, 2–6 cm. in diameter, pedunculated, fissured and grey. Two of these rats with larger tumours had widespread peritoneal metastases (Fig. 6).

Microscopically all 24 animals showed hyperplasia and hyperkeratosis of the epithelium and the small white nodules consisted of epithelium and keratin without a central core of fibrous tissue. In a few areas the base of the epithelium was also

---

EXPLANATION OF PLATES

FIG. 2 —Rat died 26 weeks after single oral dose of nitrosomethylurea (90 mg./kg. body wt.). Kidney tumour *in situ*.

FIG. 3.—Rat killed 52 weeks after nitrosomethylurea. Kidney tumour showing an area of collagen with some isolated glomeruli and tubules. H. and E. × 72.

FIG. 4.—Rat killed 52 weeks after nitrosomethylurea. Kidney tumour showing an area of elongated invasive cells. H. and E. × 290.

FIG. 5.—Rat killed 52 weeks after nitrosomethylurea. Kidney tumour showing an area of cyst formation. H. and E. × 72.

FIG. 6.—Rat died 34 weeks after oral dose of nitrosomethylurea. Carcinoma of the squamous stomach with abdominal metastases.

FIG. 7.—Rat killed 36 weeks after nitrosomethylurea. Metastatic nodule of squamous cells from stomach tumour invading the serosa and muscle layers of large intestine. H. and E. × 55.

FIG. 8.—Rat killed 42 weeks after nitrosomethylurea. Adenomas of the small and large intestine and an early kidney tumour.

FIG. 9.—Rat killed 56 weeks after nitrosomethylurea. Adenoma of the small intestine. H. and E. × 55.

FIG. 10.—Rat killed 52 weeks after nitrosomethylurea. Keratoacanthoma of the skin showing cyst formation and tongue-like projections of epithelium. H. and E. × 14.

FIG. 11.—Rat killed 51 weeks after nitrosomethylurea. Odontoma. H. and E. × 55

2

3

4

5

Leaver, Swann and Magee.

6

7

Leaver, Swann and Magee.

8

9

10

11

Leaver, Swann and Magee.

irregular and small tongues of epithelial tissue penetrated into the lamina propria. All of the small tumours and one of the large tumours were papillomas. The three other larger tumours were squamous cell carcinomas. The masses in the peritoneum of two of these rats were metastases of the primary squamous cell carcinomas (Fig. 7).

*Small intestine tumours.*—The first tumour of the small intestine was found in a rat that died at 39 weeks. Tumours were found either in the duodenum and proximal jejunum, or otherwise in the terminal ileum within 3 inches of the ileocaecal junction. There were no tumours in the middle part of the small intestine. These tumours were usually smooth, round and pedunculated and measured from 0·5–2 cm. in diameter (Fig. 8). Usually they occurred singly but in two rats two tumours were present.

Microscopically these tumours were adenomas (Fig. 9).

*Large intestine tumours.*—The first tumour of the large intestine was found in a rat that died at 24 weeks. Sixteen rats had tumours of the large intestine and in six of these the tumours were multiple. Their macroscopic and microscopic appearance closely resembled the adenomas of the small intestine. Tumours near the anus were red and haemorrhagic and fresh blood was often found in the faeces just before the animals died or were killed.

*Skin tumours.*—The first skin tumour to be recognised as such was found in a rat that died 42 weeks after treatment. They were always multiple firm nodules, which measured from 1–4 cm. in diameter and were freely moveable with the skin. Some were ulcerated at the surface. Microscopically they usually consisted of cyst-like structures situated in the dermis, containing structureless material probably derived from sebaceous glands and keratin. The lining epithelium was hyperplastic with long tongue-like projections into the lumen of the cyst (Fig. 10). These tumours were diagnosed as keratocanthomas (Ghadially, 1958, 1959; Della Porta *et al.*, 1960). In other lesions cysts had not formed but the epithelium was thickened, showed hyperkeratosis and many tongue-like projections. The ulcerated tumours showed varying degrees of necrosis of the epithelium and necrotic areas were infiltrated with inflammatory cells.

*Miscellaneous tumours.*—One rat had a lymphoma of the spleen. The spleen retained its normal shape and colour but was 5 inches in length and weighed 38 g. Two rats had tumours of the mandible involving the central part of the bone. Both tumours consisted of irregular masses of dentine and enamel associated with which was a proliferation of connective tissue which appeared similar to a fibrosarcoma (Fig. 11). These tumours were classified as odontomas since they resembled the odontomas previously described in the rat (Bullock and Curtis, 1930).

*Experiment 2: development of tumours*

In Experiment 2, groups of four rats were killed at approximately monthly intervals between 6 and 29 weeks after the dose of nitrosomethylurea. The tumours found were of the same histological type as those described in Experiment 1. No exhaustive microscopic examination was made but the only pathological change discovered other than the tumours was a marked hyperkeratosis of the squamous stomach which was found in all the animals and a cystic lesion, possibly unconnected with the experiment, in the kidney of two rats. Table II gives a list of the tumours which were found. The first tumours of the small intestine

TABLE II.—*Experiment 2: Lesions Found in Rats at Increasing Intervals After a Single Oral Dose of N-nitrosomethylurea (90 mg./kg. body wt.)*

| *Weeks after dose* | 6 | 10 | 14 | 19 | 20 | 26 | 29 |
|---|---|---|---|---|---|---|---|
| *No. of rats* | 4 | 4 | 4 | 4 | 4 | 3 | 4 |
| Squamous stomach: | | | | | | | |
| Hyperkeratosis | 4 | 4 | 4 | 4 | 4 | 3 | 4 |
| Pinhead nodules | 2 | 4 | 4 | 4 | 4 | 3 | 4 |
| Tumours | — | 1 (papilloma) | — | — | — | 1 (carcinoma) | 1 (papilloma) |
| Small intestine: | | | | | | | |
| Duodenal adenomas | — | 1 | — | — | — | 1 | 1 |
| Terminal ileum adenomas | — | — | — | — | — | — | 1 |
| Large intestine: | | | | | | | |
| Adenomas | — | — | — | — | 2 | 3 | 3 |
| Skin: | | | | | | | |
| Keratoacanthomas | — | — | — | — | 2 | 3 | 2 |
| Kidney: | | | | | | | |
| Interstitial tumours | — | — | — | — | 3 | — | 4 |

and squamous stomach were diagnosed only 10 weeks after the dose. Macroscopic tumours of the large intestine and skin were found after 20 weeks. Although the animals were flayed and the skins carefully examined, no tumours were found before this time. Tumours of the kidney were found on microscopic examination after 20 weeks, but the first macroscopic kidney tumours did not appear until 29 weeks after the dose.

*Experiment 3: early lesions*

*General features.*—The majority of early deaths occurred between the 7th and 18th days after treatment. Clinically these rats became ill after 7 days with loss of appetite and condition, they were loth to move and became quite pale. At post-mortem examination the thymus was small and grey and the mesenteric lymph nodes were haemorrhagic and oedematous in all animals. Sometimes the stomach and small intestine contained a little free blood. In most rats the mucous membrane of the squamous stomach was white and thickened, the spleen was pale and slightly shrunken and the kidneys and liver were pale. In six rats the liver was enlarged and friable with multiple white foci throughout the parenchyma. In two of these animals the myocardium contained white areas and there was a marked accumulation of fluid in the pericardial sac.

In those rats killed at specific time intervals the most obvious changes were in thymus and mesenteric lymph nodes. At 24 hours the thymus was slightly shrunken, but by 48 hours it was grey and greatly reduced in size. There was no further change until 3 weeks, when the thymus was white and about half the normal size; in rats killed at 6 and 10 weeks after treatment the thymus appeared normal. The mesenteric lymph nodes were swollen and oedematous at 48 hours, and by 7 days they were congested. At 3 weeks they were still enlarged, but by 6–10 weeks they appeared normal. The spleen was slightly shrunken and pale and the liver and kidney became pale 48 hours after dosing and remained so for 3 weeks. However, none of these rats had liver or heart lesions.

*Changes in blood and bone marrow.*—The total white cell counts are shown in Fig. 12. By 48 hours the total counts were markedly decreased and this leucopenia persisted until 2 weeks. By 3 weeks the total white cell count was within normal limits. The differential count was not significantly altered throughout the period. The total red cells and haemoglobin were decreased only at 14 days, although the reticulocyte count was increased at 4 days and remained high until 3 weeks after treatment.

In the bone marrow there was a marked reduction in all cell types at 2 days. At 4 days there was an equal proportion of juvenile erythropoietic and granulocytopoietic cells and this type of cell population persisted until 2 weeks when more mature cells began to reappear; by 3 weeks the cell population appeared normal.

FIG. 12.—Changes in total red and white blood cell counts in rats killed at regular intervals between 2 days and 3 weeks after single oral dose of nitrosomethylurea. Each point represents three rats. Dosed animals are represented thus: White cell count △——△; haemoglobin □——□. Control animals: White cell count ×---×; haemoglobin ○---○.

*Microscopic changes in rats that were killed.*—In the thymus the first changes were observed at 4 hours, when many mature lymphocytes were dark and shrunken and the pale medullary areas appeared more extensive. At 24 hours these changes were more marked and the cortex contained a large number of pale foci apparently similar to the " pock marks " described in the irradiated thymus (Murray, 1948). Each of these pale foci consisted of a large rounded macrophage with a varying amount of debris in the cytoplasm. By 72 hours the structural pattern of the thymus was reversed, so that the cortex appeared pale and the medulla dark. However, not all rats were so severely affected, and in some there was only an extension of the pale medullary area and a narrow rim of lymphocytes persisted. At 7 days mature lymphocytes had started to repopulate the cortex, and this repopulation was more extensive at 14 days. By 3 weeks the normal histological appearance of the thymus was restored. In the spleen the pattern of change closely resembled that in the thymus. There was an initial phase of lymphocyte destruction and by 4 days the follicles of the white pulp were markedly decreased in size. Repopulation by lymphocytes had commenced at 7 days and by 3 weeks the histological structure was normal. The mesenteric lymph nodes showed the same pattern of lymphocytic destruction and repopulation, but the damage was more extensive and the loss of follicles more obvious than in the spleen and thymus. In addition, between 24 hours and 7 days there was marked oedema and congestion, particularly in the medullary areas, and a few haemorrhages were also present.

The squamous stomach showed marked oedema of the subepithelial tissue at 2 days, and by 4 days the epithelium was thickened. By 7 days the oedema had resolved but the hyperkeratosis and thickening of the epithelium were more pronounced and in certain areas the basal layer was irregular with small projections into the lamina propria. At 2 weeks the epithelium was more irregular with some thickened areas and other parts appeared normal. Isolated small mucosal ulcers were present containing a small number of degenerating cells but no inflammatory cells. At the surface of the keratin layer of the mucosa there was a pronounced blue line which consisted of a heavy growth of bacteria. The epithelium remained abnormal and small white keratinising nodules were obvious at 2 weeks.

In the small intestine damage was apparent at 4 hours, when many of the cells lining the crypts showed nuclear pyknosis with the formation of a vacuole around the nucleus. By 24 hours cell debris was apparent in the lumen of the crypt, but at 48 hours the crypt epithelium was regenerating and many cells were in mitosis. At 4 days the villi appeared stunted but the degree of stunting was not assessed quantitatively. At 7 days the cells at the base of the crypts were normal and by 2 weeks the height of the intestinal epithelium appeared to have increased.

The testes were not studied systematically and special fixatives were not used. There was evidence of damage and regeneration in some tubules.

*Microscopic changes in rats that died.*—The pattern of change in these rats was similar to those that were killed but the degree of damage, particularly to the thymus, spleen and mesenteric lymph nodes, was more extensive. The white patches observed macroscopically in the heart were myocardial degeneration with replacement fibrosis and the liver lesions were areas of necrosis resembling small infarcts, randomly distributed throughout the parenchyma. Inflammatory cells were absent from both the heart and the liver lesions.

### DISCUSSION

In these experiments nitrosomethylurea proved carcinogenic for the rat when given as a single oral dose of 90 mg./kg. body wt. The induction of tumours in the kidney substantiates the prediction based on previous biochemical work on methylation of nucleic acids in this organ by nitrosomethylurea and dimethylnitrosamine (Swann and Magee, 1968).

Nitrosomethylurea is unstable in aqueous solution and unlike dimethylnitrosamine may not require enzymic conversion to an active proximal carcinogen. The activity of the compound as a topical skin carcinogen (Graffi *et al.*, 1967) and the wide distribution of primary tumours found after intragastric administration in these experiments, and after intravenous injection in the experiments of Druckrey *et al.* (1964) would be consistent with this conclusion.

In correlating the initial pathological changes with the subsequent induction of tumours in the same organ, there appeared to be little association between either the initial occurrence of cell necrosis, or the rapidity of cell turnover in an organ, and the subsequent development of tumours. Thus, although the epithelium of the squamous stomach was altered within 4 days, necrosis was not marked and yet subsequently papillomas and squamous cell carcinomas developed. Toledo (1965) reported similar findings with the chemically related carcinogen N-nitrosomethylurethane. Administering highly diluted solutions of this compound he concluded that preceding necrosis is not obligatory for the development of hyperplasia or papilloma in rat stomach. Furthermore, necrosis was not observed in the kidney even though 50% of the rats eventually developed renal tumours. The kidney, with a relatively slow cell turnover, developed tumours as readily as the small and large intestine which have a rapid cell turnover.

The kidney tumours varied considerably in structure and were similar to those produced by dimethylnitrosamine (Magee and Barnes, 1959, 1962; Riopelle and Jasmin, 1963; Thomas and Schmähl, 1964) and by the naturally-occurring carcinogen cycasin (Laqueur *et al.*, 1963; Laqueur and Matsumoto, 1966; Gusek *et al.*, 1966). These tumours have been variously described as anaplastic, mesenchymal, interstitial, sarcoma and nephroblastoma.

The induction of skin tumours in the rat after a single oral dose of carcinogen is unusual and the development of keratoacanthomas rather than squamous papillomas and carcinomas, as reported by Graffi *et al.* (1967) may reflect the arrival of the carcinogen via the blood stream rather than by topical application. On the other hand, diazoacetic ester induced squamous papillomas and carcinomas of the skin in rats after intravenous injections (Druckrey *et al.*, 1965). The two lesions of the mandible were apparently similar to two described by Bullock and Curtis (1930) as odontomas, occurring spontaneously in the rat. The rarity of this tumour suggests that those described in this experiment were produced by the carcinogen. This conclusion is supported by the recent reports by Druckrey (1967) of an odontoblastoma following a single intravenous injection of nitrosomethylurea in the rat and by Herrold (1968) of the induction of odontogenic tumours in the Syrian golden hamster by repeated intravenous or intragastric administration of nitrosomethylurea.

From the observations on rats killed at regular intervals between 6 and 29 weeks after treatment (Experiment 2) it seems that there may be a variable period when growth of the tumours is not apparent followed by a period of rapid tumour growth. In both Experiment 2 and Experiment 1 the first neoplasms were discovered in the stomach and small intestine 10 weeks after dosing. The tumours of the skin and kidney did not appear until much later. The first kidney tumours were discovered on microscopic examination 20 weeks after the dose and only became visible macroscopically after 29 weeks. The first skin tumours were not detected until 20 weeks.

The early action of nitrosomethylurea is similar to that of many other biological alkylating agents (Dustin, 1947; Boyland, 1954; Clayson 1962), with cytotoxicity in organs such as the bone marrow and intestinal epithelium which have rapid cell division. The liver and heart lesions occurring 7 to 10 days after treament resembled anaemic infarcts but their exact pathogenesis is not clear. Some of the liver lesions and one heart lesion were cultured for bacteria without success and there was no evidence of bacteria in the stained sections. Whatever the true nature of these lesions, it is unlikely that they were caused by a direct toxic effect of nitrosomethylurea, because of their delayed appearance and their occurrence in only some of the rats.

SUMMARY

N-methyl-N-nitrosourea was given in a single intragastric dose of 90 mg./kg. body wt to three groups of young Wistar rats, since previous biochemical experiments had led to the prediction that this dose would produce a high incidence of tumours of the kidney. Rats from the first group were kept until they died or were obviously ill. Thirty-one survived more than 1 month. Tumours were found in the kidney (15), small intestine (6), large intestine (16), jaw (odontoma) (2), and skin (keratoacanthoma) (8). In the squamous stomach, tumours greater than 3 mm. diameter were found in nine rats; in 15 other animals numerous small white nodules were found on the squamous mucosa. The microscopic appearance of the tumours is described. Rats from the second experiment were killed in groups of four at intervals from 6 to 29 weeks after the dose. The first tumours of the stomach and small intestine were found 10 weeks after the dose. Tumours of the large intestine, skin and kidney were found 20 weeks after the dose. Rats killed at intervals between 2 hours and 3 weeks after the dose showed pathological change particularly in the thymus, lymph nodes and bone marow. There was a marked reduction of all cell types in the bone marrow and an acute leucopoenia persisted for 2 weeks after the dose. The results of a general histological survey are reported.

We are very grateful to Dr. W. H. Butler and Dr. B. Terracini for their advice on the classification of some of the pathological specimens, and to Mr. I. F. Gaunt, B.Sc., M.I.Biol., of the British Industrial Biological Research Association, for the blood and marrow counts, and Mr. R. F. Legg, A.I.M.L.T., who prepared the photomicrographs.

### REFERENCES

Boyland, E.—(1954) *Pharmac. Rev.*, **6**, 345.

Bruce, H. M. and Parkes, A. S.—(1956) *J. Anim. Techns Ass.*, **7**, 54.

Bullock, F. D. and Curtis, M. R.—(1930) *J. Cancer Res.*, **14**, 1.

Clayson, D. B.—(1962) ' Chemical Carcinogenesis ', London (J. and A. Churchill).

Craddock, V. M. and Magee, P. N.—(1963) *Biochem. J.*, **89**, 32.

Della Porta, G., Terracini, B., Dammert, K. and Shubik, P.—(1960) *J. natn. Cancer Inst.*, **25**, 573.

Druckrey, H.—(1967) *In*: ' Potential Carcinogenic Hazards from Drugs '. Edited by Truhaut, R. Berlin, Heidelberg and New York (Springer-Verlag) p. 60.

Druckrey, H., Ivankovic, S. and Preussmann, R.—(1965) *Z. Krebsforsch.*, **66**, 389.

Druckrey, H., Ivankovic, S. and So, B. T.—(1965) *Z. Krebsforsch.*, **66**, 523.

Druckrey, H., Preussmann, R., Ivankovic, S. and Schmähl, D.—(1967) *Z. Krebsforsch.*, **69**, 103.

Druckrey, H., Preussmann, R., Schmähl, D. and Müller, M.—(1961) *Naturwissenschaften*, **48**, 165.

Druckrey, H., Steinhoff, D., Preussmann, R. and Ivankovic, S.—(1964) *Z. Krebsforsch.*, **66**, 1.

Dustin, P.—(1947) *Nature, Lond.*, **159**, 794.

Ghadially, F. N.—(1958) *J. Path. Bact.*, **75**, 441.—(1959) *J. Path. Bact.*, **77**, 277.

Graffi, A., Hoffmann, F. and Schütt, M.—(1967) *Nature, Lond.*, **214**, 611.

Gusek, W., Buss, H. and Kruger, C. H.—(1966) *Verh. dt. Ges. Path.*, **50**, 337.

Herrold, K. M.—(1966) *J. Path. Bact.*, **92**, 35.—(1968) *Oral Surg.*, **25**, 262.

Jänisch, W. and Schreiber, D.—(1967) *Naturwissenschaften*, **54**, 171.

Jänisch, W., Schreiber, D., Stengel, R. and Steffen, V.—(1967) *Expl Path.*, **1**, 243.

Laqueur, G. L. and Matsumoto, H.—(1966) *J. natn. Cancer Inst.*, **37**, 217.

LAQUEUR, G. L., MICKELSEN, O., WHITING, M. G. AND KURLAND, L. T.—(1963) *J. natn. Cancer Inst.*, **31**, 919.
LEE, K. Y., LIJINSKY, W. AND MAGEE, P. N.—(1964) *J. natn. Cancer Inst.*, **32**, 65.
MAGEE, P. N. AND BARNES, J. M.—(1959) *Acta Un. int. Cancr.*, **15**, 187.—(1962) *J. Path. Bact.*, **84**, 19.—(1967) *Adv. Cancer Res.*, **10**, 163.
MAGEE, P. N. AND FARBER, E.—(1962) *Biochem. J.*, **83**, 114.
MAGEE, P. N. AND HULTIN, T.—(1962) *Biochem. J.*, **83**, 106.
MAGEE, P. N. AND SCHOENTAL, R.—(1964) *Br. med. Bull.*, **20**, 102.
MURRAY, R. G.—(1948) *In*: ' Histopathology of Irradiation from External and Internal Sources '. Edited by Bloom, W. New York, Toronto and London (McGraw-Hill) p. 446.
RIOPELLE, J. L. AND JASMIN, G.—(1963) *Revue can. Biol.*, **22**, 365.
SCHREIBER, D. AND JÄNISCH, W.—(1967) *Expl Path.*, **1**, 331.
SWANN, P. F. AND MAGEE, P. N.—(1968) *Biochem. J.*, **110**, 39.
THOMAS, C. AND SCHMÄHL, D.—(1964) *Z. Krebsforsch.*, **66**, 125.
THOMAS, C., SIERRA, J. L. AND KERSTING, G.—(1967) *Naturwissenschaften*, **54**, 228.
TOLEDO, J. D.—(1965) *Beitr. path. Anat.*, **131**, 63.
WERNER, E. A.—(1919) *J. chem. Soc.*, **115**, 1093.